# Topologically Disrupted Gray Matter Networks in Drug-Naïve Essential Tremor Patients With Poor Sleep Quality

**DOI:** 10.3389/fneur.2022.834277

**Published:** 2022-04-26

**Authors:** Jiaxin Peng, Jing Yang, Nannan Li, Du Lei, Junying Li, Liren Duan, Chaolan Chen, Yan Zeng, Jing Xi, Yi Jiang, Qiyong Gong, Rong Peng

**Affiliations:** ^1^Department of Neurology, West China Hospital, Sichuan University, Chengdu, China; ^2^Department of Radiology, Huaxi MR Research Center (HMRRC), West China Hospital, Sichuan University, Chengdu, China

**Keywords:** essential tremor, poor quality of sleep, gray matter network, graph theory, brain network

## Abstract

**Background:**

Sleep disturbances are widespread among patients with essential tremor (ET) and may have adverse effects on patients' quality of life. However, the pathophysiology underlying poor quality of sleep (QoS) in patients with ET remains unclear. Our study aimed to identify gray matter (GM) network alterations in the topological properties of structural MRI related to QoS in patients with ET.

**Method:**

We enrolled 45 ET patients with poor QoS (SleET), 59 ET patients with normal QoS (NorET), and 66 healthy controls (HC), and they all underwent a three-dimensional T1-weighted MRI scan. We used a graph-theoretical approach to investigate the topological organization of GM morphological networks, and individual morphological brain networks were constructed according to the interregional similarity of GM volume distributions. Furthermore, we performed network-based statistics, and partial correlation analyses between topographic features and clinical characteristics were conducted.

**Results:**

Global network organization was disrupted in patients with ET. Compared with the NorET group, the SleET group exhibited disrupted topological GM network organization with a shift toward randomization. Moreover, they showed altered nodal centralities in mainly the frontal, temporal, parietal, and cerebellar lobes. Morphological connection alterations within the default mode network (DMN), salience, and basal ganglia networks were observed in the SleET group and were generally more extensive than those in the NorET and HC groups. Alterations within the cerebello-thalamo-(cortical) network were only detected in the SleET group. The nodal degree of the left thalamus was negatively correlated with the Fahn-Tolosa-Marin Tremor Rating Scale score (*r* = −0.354, *p* =0.027).

**Conclusion:**

Our findings suggest that potential complex interactions underlie tremor and sleep disruptions in patients with ET. Disruptions within the DMN and the cerebello-thalamo-(cortical) network may have a broader impact on sleep quality in patients with ET. Our results offer valuable insight into the neural mechanisms underlying poor QoS in patients with ET.

## Introduction

Essential tremor (ET) is one of the most common movement disorders, with an overall prevalence of ~0.32–1% in the general population (1). The traditional conception of ET as a mono-symptomatic disorder has been challenged by growing evidence of various additional neurological signs and non-motor symptoms that accompany ET. Among the non-motor symptoms associated with ET, sleep disturbances have gained increased attention and include increased daytime sleepiness (2) and decreased nighttime sleep quality (3) in patients with ET compared with healthy controls (HCs). Poor quality of sleep (QoS) is often distressing and impairs the quality of life of patients with ET. However, previous studies concerning poor QoS in patients with ET have mainly focused on the comparisons of symptoms (3, 4), and the neural substrates of poor QoS have not yet been investigated. Thus, whether neural changes underlying ET are precursors to the development of poor QoS remains unknown. Therefore, investigations of brain morphological changes would provide evidence of the interactions of ET and poor QoS and broaden our knowledge of the neural mechanisms underlying the heterogeneity among patients with ET.

Neuroimaging, especially MRI, has been widely utilized to investigate functional and morphological connectivity alterations in patients with ET. Knowledge of the neural basis of ET outside the traditional “tremor network,” also known as the cerebello-thalamocortical network (5), has improved considerably over the past 5 years. Evidence from structural MRI has demonstrated that specific damage to motor network structures, such as the supplementary motor area (SMA), and alterations in the integrity of frontal and parietal areas contribute to the heterogeneity of ET motor symptoms (6, 7). Furthermore, patients with ET exhibit widespread disrupted brain functional networks, which include those irrelevant to tremors (8). In our previous study, we found gray matter (GM) dysconnectivity within the frontal, parietal, and cerebellar lobes in drug-naive patients with ET based on T1-weighted MRI (9). Moreover, MRI studies have shown that poor QoS in healthy adults is associated with higher amyloid burden, brain cortical thinning, and functional/structural connectivity alterations of distinct networks, especially the default mode network (DMN) (10–12). These findings indicate progress in understanding the role of QoS in disease and suggest potential interference of poor QoS in patients with ET.

The graph theory approach models the human brain as a complex network that is constructed by nodes and edges to create a delicate mathematical framework to characterize the topological organization of the human brain (13). Based on this framework, the human brain exhibits a “small world” organization, which allows the optimal balance between the segregation and integration of information processing (14). By using this method, previous studies have identified large-scale topological dysconnections of functional networks in patients with ET (8, 15), although only one study has explored GM morphological networks (9). We used a new method proposed by Kong et al. (16) to construct a GM structural covariance network, based on the graph theory approach using structural MRI, by computing morphological similarity relationships of GM at the individual level (17). This approach can quantify the interregional relationships within each patient's brain and also estimate the structural complexity of the cerebral cortex. Therefore, applying this promising approach to investigations of morphological networks could provide new insights into the causes and interference of whole-brain GM network alterations in patients with ET having poor QoS.

This study is the first structural MRI study using the graph theory approach to investigate brain morphological network alterations in patients with ET having poor QoS. We aimed to identify structural connectome alterations in the topological properties of T1-weighted MRI to evaluate the effect of subjective poor QoS in patients with ET and explore the underlying neural bases. We hypothesized that patients with ET would show disrupted topological organization in GM morphological networks in comparison with HCs, and the extent of disruption would be greater in patients with ET having poorer QoS (SleET). In addition, given reports demonstrating the vital role of the DMN in QoS (18) and the potential association between tremor and the cerebello-thalamocortical network (5), we hypothesized that nodes involving the DMN and the cerebello-thalamocortical network would exhibit altered nodal topological properties in patients in the SleET group compared with patients with ET having normal QoS (NorET) and HCs.

## Materials and Methods

### Participants

Patients with ET were consecutively recruited from the outpatient clinic of the Neurology Department of West China Hospital, Sichuan University, from July 2015 to June 2021. The study was approved by the local ethics committee of West China Hospital, and all participants provided written informed consent before enrollment. The diagnosis of ET was based on the Consensus Criteria of Clinical Diagnosis of Essential Tremor of the Movement Disorder Society (MDS) 1998 (19). Participants were then screened for eligibility according to the following inclusion/exclusion criteria. The inclusion criteria for patients with ET were the following: (a) postural and/or kinetic tremor involving both upper extremities; (b) disease duration of 3 years or more; (c) aged between 20 and 80 years; (d) right-handedness. Right-handed, age-, and sex-matched healthy controls were recruited through poster advertisements or unrelated family members or friends of patients. The exclusion criteria for all participants were the following: (a) identifiable brain lesions on T1- or T2-weighted MRI; (b) presence of head movement artifacts on scanning; (c) patients with dystonia or bradykinesia related to Parkinsonism; (d) presence of other neurological signs that indicate secondary tremor disorders; (e) obvious stepwise tremor progression; (f) history of psychological disorders or dementia; (g) obvious symptoms of depression/anxiety; (h) traumatic or stressful life events within the last year; (i) history of receiving anti-dementia, antidepressant, anticholinergic, sedative, or anti-tremor medications (including beta-blockers and gamma-aminobutyric acid [GABA] derivatives). Demographic information (including sex, age, and years of education) was collected and basic blood tests were performed during the first visit. Clinical evaluations were then performed during face-to-face interviews on the same day before the scheduled MRI examination. All assessment was performed before patients were administered medication treatments for tremor and sleep disturbances to exclude any confounders of medication.

We used the Fahn-Tolosa-Marin Tremor Rating Scale (TRS) (20) to assess tremor severity (the TRS contains TRS-A, TRS-B, and TRS-C domains). The Pittsburg Sleep Quality Index (PSQI) (21) was used to evaluate subjective QoS. ET patients with a PSQI score of ≥6 were regarded as having poor QoS (SleET), and those with a PSQI score of <6 were regarded as having normal QoS (NorET). In addition, we used the Mini-Mental State Examination (MMSE) (22) to evaluate cognitive function and excluded those with an MMSE score <24, which indicated mild cognitive impairment. The Hamilton Anxiety Scale (HAMA) (23) and the Hamilton Depression Scale (HAMD-24) (24) were used to assess mood symptoms, and we excluded participants with a HAMA score of >14 or a HAMD score of >17 (25), which indicated symptoms of anxiety or depression, respectively. The final sample comprised 45 patients in the SleET group, 59 patients in the NorET group, and 66 HCs.

### Data Acquisition and Data Processing

All participants underwent an MRI scan on the same scanner (3 T Siemens Trio, Erlangen, Germany) using the same sequence and an eight-channel phased-array head coil. Whole-brain high-resolution three-dimensional (3D) T1-weighted images were acquired with the following parameters: echo time (TE) 2.26 ms, repetition time (TR) 1,900 ms, inversion time 900 ms, flip angle 9°, slice thickness 1 mm, no inter-slice gap, single excitation, field of view 256 mm × 256 mm, voxel size 1 m × 1 m × 1 mm, 176 sagittal slices, and matrix size 176 × 202 × 200. Participants' heads were immobilized using foam pads to minimize motion artifacts. We manually verified the quality of the raw MRI images and evaluated clinical abnormalities in a double-blinded manner.

Data processing was performed using the SPM12 software (http://www.fil.ion.ucl.ac.uk/spm/software/spm12/) in the MATLAB 2013b software environment (MathWorks, Natick, MA, United States). Processing steps included the following: (a) conversion of DICOM data into NIFTI data; (b) segmentation of 3D T1-weighted MRI images to obtain GM images; (c) manual checking of image quality; (d) using the Diffeomorphic Anatomical Registration through Exponential Lie Algebra (DARTEL) tool within the SPM12 package to create a custom template from related tissue segments and performing nonlinear transformation of the original image to the normalized image; (e) conversion of the segmented image to Montreal Neurological Institute stereotactic space; (f) resampling GM images into 2 mm × 2 mm × 2 mm voxels; (g) spatial smoothing of GM images using a 6 mm full-width at half-maximum Gaussian kernel.

### Network Construction

Network construction was performed in MATLAB 2013b. A critical task for constructing the human brain network is defining the nodes and edges. First, the whole-brain GM image was divided according to the automated anatomical labeling (AAL) algorithm nodes into 116 regions of interest (ROIs), which were defined as the nodes. We then extracted GM volume (GMV) values for all the voxels within each ROI. The probability density functions (PDFs) of the GMV values were estimated using kernel density estimation (26). The details of the analyses are described elsewhere (16, 27). Kullback-Leibler divergence-based similarity (KLS) values (possible KLS values range from 0 to 1, with one representing two identical distributions) (17) were calculated between all possible pairs of ROIs using their PDFs. The network edges were then defined as interregional connections based on the quantified morphological similarity between two ROIs. Finally, a KLS-based 116 × 116 morphological connection matrix was generated for each participant.

### Network Properties

The calculation of GM network properties was performed in the MATLAB 2013b software environment using the Graph Theoretical Network Analysis (GRETNA) graph-based network analysis toolkit (http://www.nitrc.org/projects/Gretna/) (28). A wide range of sparsity (S) thresholds was applied to each correlation matrix to ensure that the thresholded networks were estimable for small-worldness with sparse properties and minimum spurious edges (29). Thus, we calculated both global and nodal network metrics at *S* thresholds ranging from 0.1 to 0.35, with an interval of 0.01. We then calculated the area under the curve (AUC) for the above *S* range, in line with previous studies (30, 31), to characterize brain networks that were free of the potential bias introduced by any single threshold. The properties of the GM network at each *S* level were calculated using the following parameters: (1) global network measures, including network efficiency (i.e., global efficiency [*E*_global_] and local efficiency [*E*_local_]), the clustering coefficient (*C*_p_), characteristic path length (*L*_p_), normalized clustering coefficient (γ), normalized characteristic path length (λ), and small-worldness (σ); (2) nodal centrality measures for each node, including nodal degree, nodal betweenness, and nodal efficiency.

### Statistical Analysis

Demographics and clinical characteristics were analyzed using SPSS 24 (IBM Corporation, Armonk, NY, United States). Continuous variables were analyzed using a univariate one-way ANOVA, followed by *post-hoc t*-tests between each pair of groups. The continuous variables were compared between the two ET groups using two-sample *t*-tests. Categorical variables were analyzed using chi-squared tests. We then used nonparametric permutation tests for the AUC of each network measure to detect significant differences among the three groups (32). *Post-hoc* pairwise permutation tests were conducted for measures with significant group differences. The randomization was repeated 10,000 times. the false discovery rate (FDR) correction method was applied for multiple comparisons at a significance level of 0.05 (33).

Region pairs with between-group differences in nodal properties were identified using the network-based statistics (NBS) method (http://www.nitrc.org/projects/nbs/) (University of Melbourne, Melbourne, Victoria, Australia) (34). We included nodes that exhibited significant between-group differences (*p* < 0.05, FDR corrected). First, a one-way ANOVA was performed to define a set of significant changes between the connected regions (*p* < 0.05, threshold *F* = 4.64). Then, *post-hoc t*-tests were performed between each pair of subgroups (*p* < 0.05, threshold *T* = 2.64). All connections were then tested for significance using a nonparametric permutation method with 10,000 permutations.

Finally, we conducted partial correlations to explore the relationships between significant GM network values and clinical characteristics, with age, sex, years of education, HAMA score, and HAMD score as covariates. The clinical variables included were the age of onset, disease duration, TRS score, and PSQI score.

## Results

### Demographic and Clinical Characteristics

The final sample comprised 45 patients in the SleET group, 59 patients in the NorET group, and 66 HCs. The demographic and clinical data are summarized in Table 1. No significant differences were found among the three groups for age, sex, or years of education (*p* > 0.05). The SleET group were older at age of onset (*p* = 0.001) and had higher TRS (*p* = 0.012), TRS-B (*p* = 0.006), HAMA (*p* < 0.001), and HAMD scores (*p* < 0.001) than the NorET group. There were no significant differences between the SleET and NorET groups for disease duration, tremor distribution, tremor type, tremor asymmetry, or MMSE score (*p* > 0.05).

**Table 1:** Demographic and clinical characteristics of the patients having poor quality of sleep (QoS; SleET), patients having normal QoS (NorET), and healthy control (HC) participants.

	**SleET**	**NorET**	**HC**	***p***	
				**ANOVA**	**Sle-ET vs. Nor-ET**
Age	54.29 ± 14.783	53.69 ± 14.695	52.55 ± 11.169	0.163	0.221
Sex (M/F)	13/32	20/39	21/45	0.076	0.064
Years of Education	10.27 ± 4.059	11.36 ± 4.246	10.583 ± 3.914	0.091	0.135
Handedness (Right/Left)	45/0	59/0	66/0	>0.999	>0.999
Age of onset	42.98 ± 16.876	31.56 ± 16.424	–	–	**0.001**
Disease duration	11.800 ± 10.612	11.856 ± 9.952	–	–	0.978
Positive family History	19 (42.2%)	29 (49.2%)	–	–	0.558
**Tremor distribution**					
Upper limbs	45 (100%)	59 (100%)	–	–	> 0.999
Head	16 (41.0%)	12 (23.5%)	–	–	0.097
Voice/Tongue/face	9 (25.7%)	13 (28.3%)	–	–	0.808
Legs	5 (14.3%)	5 (11.4%)	–	–	0.743
Trunk	1 (3.0%)	0	–	–	0.440
**Tremor type**			–	–	
Postural tremor	45 (100%)	59 (100%)	–	–	> 0.999
Kinetic tremor	13 (28.9%)	19 (32.2%)	–	–	0.831
Rest tremor	17 (37.8%)	14 (23.7%)	–	–	0.135
Intention tremor	16 (35.6%)	15 (25.4%)	–	–	0.286
**Tremor asymmetry**			–	–	
Left = Right	19 (43.2%)	35 (59.3%)	–	–	0.224
Left > Right	14 (31.8%)	11 (18.6%)	–	–	0.207
Left < Right	11 (25.0%)	13 (22.0%)	–	–	0.203
TRS	25.82 ± 16.395	17.00 ± 13.000	–	–	**0.003**
TRS-A	6.53 ± 4.916	5.31 ± 3.715	–	–	0.150
TRS-B	14.09 ± 7.885	8.61 ± 6.571	–	–	**<** **0.001**
TRS-C	5.02 ± 5.483	3.10 ± 3.759	–	–	**0.037**
MMSE	26.89 ± 3.164	27.17 ± 4.568	27.94 ± 1.788	0.219	0.725
PSQI	17.55 ± 5.509	3.91 ± 2.933	2.64 ± 2.377	**<** **0.001**	**<** **0.001**
HAMA	9.20 ± 6.147	4.66 ± 4.622	3.79 ± 3.571	**<** **0.001**	**<** **0.001**
HAMD	9.89 ± 6.147	4.25 ± 4.241	2.91 ± 2.653	**<** **0.001**	**<** **0.001**

*Bold numbers in the last column are statistically significant at p < 0.05*.

*SleET, essential tremor with poor sleep quality; NorET, essential tremor with normal sleep quality; HC, healthy controls; TRS, Fahn-Tolosa-Marin Tremor Rating Scale; MMSE, Mini-mental State Examination; PSQI, Pittsburg Sleep Quality Index; HAMA, Hamilton Anxiety Rating Scale; HAMD, Hamilton Depression Rating Scale*.

### Alterations in Global Brain Network Properties

**Figure 1:**
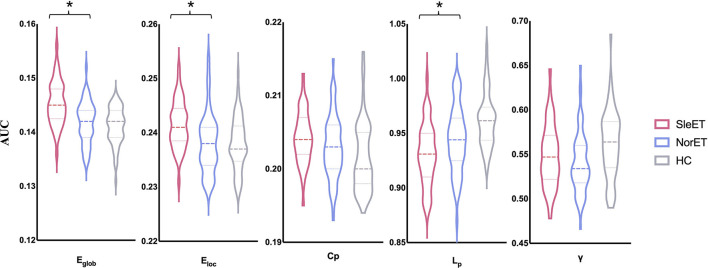
Global gray matter network properties differed significantly among the SleET, NorET, and HC groups. Black asterisks indicate significant differences in the *post-hoc* comparisons between SleET and NorET. ET, essential tremor; SleET, ET with poor sleep quality; NorET, ET with normal sleep quality; HC, healthy control; AUC, area under the curve; *E*_glob_, global efficiency; *E*_loc_, local efficiency; *C*_p_, clustering coefficient; *L*_p_, characteristic path length; γ, normalized clustering coefficient; σ, small-worldness.

All three groups exhibited λ ≈ 1 and λ > 1, which indicated small-world organization in the defined threshold range. There were significant group effects in λ, *E*_glob_, *E*_loc_, *C*_p_, and *L*_p_ among the global network measures of the three groups (Table 2; Figure 1). *Post-hoc* comparisons showed that, relative to HCs, both ET groups showed lower *L*_p_ and λ, and the SleET group showed higher *E*_glob_ (*p* < 0.001), *E*_loc_ (*p* < 0.001), and *C*_p_ (*p* = 0.0023). Moreover, relative to the NorET group, the SleET group showed higher *E*_glob_ (*p* < 0.001) and *E*_loc_ (*p* = 0.0016) and lower *L*_p_ (*p* =0.0038).

**Table 2:** Brain topological metrics showing differences among the SleET and NorET patient groups and HCs.

**Measurements**	**ANOVA *P* (*F*-values)**	***Post-hoc p (t-values)***		
		**SleET vs. NorET**	**SleET vs. HC**	**NorET vs. HC**
*E*_glob_	**0.0001 (16.065)**	**0.0001 (3.924)**	**0.0001 (5.633)**	0.0650 (1.550)
*E*_loc_	**0.0018 (6.629)**	**0.0016 (3.056)**	**0.0001 (3.504)**	0.3784 (0.315)
*C*_p_	**0.0256 (3.689)**	0.0762 (1.440)	**0.0023 (2.774)**	0.0734 (1.421)
*L*_p_	**0.0001 (15.132)**	**0.0038 (−2.697)**	**0.0001 (−5.562)**	**0.0027 (−2.878)**
γ	**0.0001 (12.034)**	0.0257 (1.964)	**0.0070 (−2.503)**	**0.0001 (−4.834)**
**Nodal degree**				
Frontal_Mid_L	**0.0001 (12.481)**	**0.0021 (−4.374)**	**0.0001 (−4.497)**	0.1889 (**–**0.9127)
Frontal_Mid_R	**0.0001 (20.883)**	**0.0001 (−4.564)**	**0.0001 (−6.509)**	0.0164 (**–**2.155)
Frontal_Inf_Tri_R	**0.0001 (71.636)**	**0.0001 (−6.002)**	**0.0001 (−8.519)**	**0.0001 (−8.534)**
Frontal_Sup_Medial_L	**0.0001 (26.360)**	**0.0001 (−5.418)**	**0.0001 (−6.780)**	0.0189 (**–**2.384)
Cingulum_Mid_L	**0.0053 (5.616)**	**0.0022 (2.376)**	**0.0019 (4.849)**	0.3698 (0.347)
SupraMarginal_L	**0.0016 (7.064)**	0.0274 (1.101)	**0.0004 (4.898)**	0.2621 (0.639)
Precuneus_R	**0.0001 (58.472)**	**0.0001 (5.665)**	**0.0001 (−4.549)**	**0.0001 (−9.938)**
Pallidum_L	**0.0001 (48.201)**	**0.0001 (−5.944)**	**0.0030 (2.727)**	**0.0001 (7.954)**
Pallidum_R	**0.0001 (24.474)**	**0.0001 (−3.765)**	**0.0028 (2.635)**	**0.0001 (6.359)**
Thalamus_L	**0.0001 (14.739)**	**0.0028 (−2.746)**	**0.0001 (−6.697)**	**0.0002 (−4.003)**
Temporal_Pole_Sup_R	**0.0001 (31.387)**	**0.0016 (3.120)**	**0.0001 (−4.418)**	**0.0001 (−7.627)**
Cerebellum_Crus2_R	**0.0006 (8.282)**	**0.0003 (2.657)**	0.0166 (1.670)	0.0225 (1.995)
Cerebellum_8_R	**0.0001 (8.688)**	0.0226 (1.899)	**0.0001 (4.613)**	**0.0006 (3.033)**
Vermis_10	**0.0001 (57.353)**	**0.0001 (5.376)**	**0.0001 (9.229)**	**0.0001 (6.878)**
**Nodal betweenness**				
Frontal_Inf_Tri_R	**0.0001 (10.041)**	**0.0036 (−2.494)**	**0.0001 (−3.945)**	**0.0041 (−2.615)**
Supp_Motor_Area_R	**0.0011 (7.367)**	**0.0019 (3.089)**	**0.0002 (3.579)**	0.3459 (0.391)
Hippocampus_L	**0.0001 (23.451)**	**0.0008 (−3.056)**	**0.0005 (3.008)**	**0.0001 (7.362)**
SupraMarginal_L	**0.0024 (5.816)**	0.0344 (2.908)	**0.0024 (3.226)**	0.3573 (0.412)
Vermis_10	**0.0001 (11.861)**	**0.0011 (3.045)**	**0.0001 (4.343)**	**0.0121 (2.221)**
**Nodal efficiency**				
Frontal_Mid_L	**0.0127 (4.486)**	0.0254 (**–**1.445)	**0.0073 (−2.721)**	0.1992 (**–**0.842)
Frontal_Mid_R	**0.0001 (12.234)**	**0.0001 (−4.978)**	**0.0008 (−3.089)**	0.0167 (**–**2.162)
Frontal_Inf_Oper_L	**0.0288 (3.678)**	0.0185 (1.339)	**0.0073 (2.386)**	0.1929 (0.869)
Frontal_Inf_Tri_R	**0.0001 (53.188)**	**0.0001 (−5.960)**	**0.0001 (−8.676)**	**0.0001 (−6.163)**
Frontal_Sup_Medial_L	**0.0001 (23.751)**	**0.0001 (−4.273)**	**0.0001 (−6.388)**	**0.0011 (−3.091)**
Cingulum_Mid_L	**0.0001 (14.691)**	**0.0001 (5.052)**	**0.0001 (5.071)**	0.4891 (0.016)
Amygdala_R	**0.0001 (30.158)**	**0.0012 (3.027)**	**0.0001 (6.124)**	**0.0001 (5.335)**
Calcarine_R	**0.0008 (7.501)**	0.0178 (1.106)	**0.0015 (3.858)**	0.2643 (0.608)
Pallidum_L	**0.0001 (53.562)**	**0.0001 (−5.195)**	**0.0001 (5.425)**	**0.0001 (9.296)**
Pallidum_R	**0.0001 (32.961)**	**0.0010 (−3.278)**	**0.0001 (5.217)**	**0.0001 (7.697)**
Thalamus_R	**0.0010 (4.946)**	0.0266 (1.773)	**0.0001 (3.612)**	0.0243 (1.986)
Temporal_Pole_Sup_R	**0.0001 (29.294)**	**0.0015 (−3.242)**	**0.0002 (3.998)**	**0.0001 (7.009)**
Cerebellum_8_R	**0.0001 (20.014)**	**0.0001 (4.182)**	**0.0001 (5.872)**	**0.0016 (3.111)**
Vermis_10	**0.0001 (46.082)**	**0.0001 (6.930)**	**0.0001 (8.534)**	**0.0008 (3.366)**

*Comparisons of global and nodal measures among the three groups (p < 0.05, shown in bold) were performed using nonparametric permutation tests*.

*SleET, essential tremor with poor sleep quality; NorET, essential tremor with normal sleep quality; HC, healthy controls; ANOVA, analysis of variance; E_glob_, global efficiency; E_loc_, local efficiency; C_p_, clustering coefficient; L_p_, characteristic path length; γ, normalized clustering coefficient; L, left; R, right; Mid, middle; Inf, inferior; Tri, triangular; Sup, superior; Post, posterior; Supp, supplementary; Oper, opercular*.

### Alterations of Nodal Brain Network Properties

**Figure 2:**
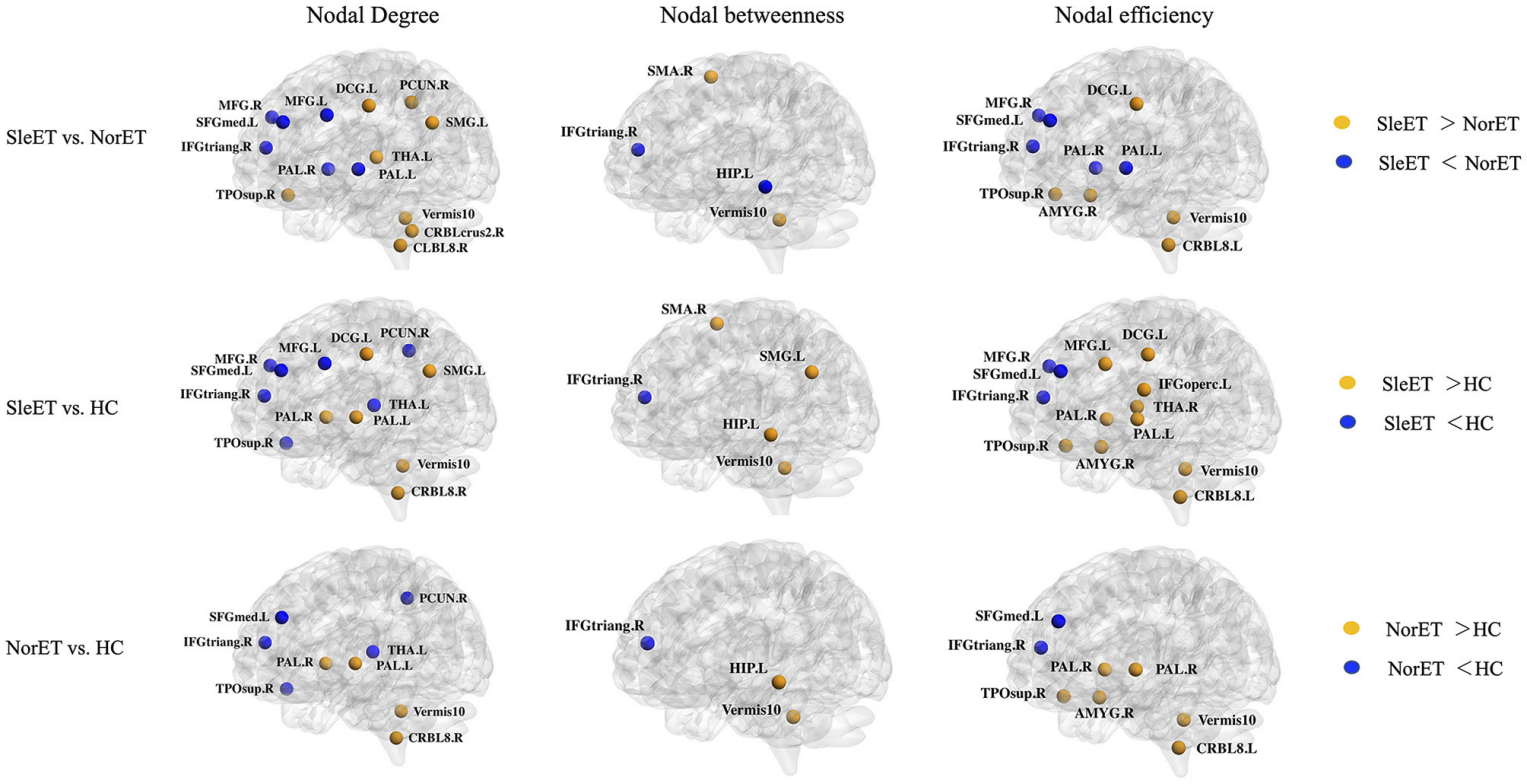
Brain regions with significant group effects in the nodal centralities of morphological brain networks compared among the SleET, NorET, and HC groups. Regions are located according to their centroid stereotaxic coordinates. ET, essential tremor; SleET, ET with poor sleep quality; NorET, ET with normal sleep quality; HC, healthy control; MFG, middle frontal gyrus; IFGtriang, inferior frontal gyrus, triangular part; SFGmed, superior frontal gyrus, medial; SMA, supplementary motor area; PCUN, precuneus; HIP, hippocampus; AMYG, amygdala; DCG, medial cingulate and paracingulate gyri; CAL, calcarine fissure; SMG, supramarginal gyrus; PAL, pallidum; THA, thalamus; CRBL, cerebellum; L, left; R, right.

Brain regions with altered nodal centrality in at least one nodal property are listed in Table 2 (FDR corrected, *p* < 0.05). *Post-hoc* comparisons showed that relative to HCs, both ET groups showed lower nodal centralities in the right triangular part of the inferior frontal gyrus (IFGtriang), left medial superior frontal gyrus (SFGmed), right precuneus (PCUN), and left thalamus (THA), and higher nodal centralities in the left hippocampus (HIP), right amygdala (AMYG), bilateral pallidum (PAL), right cerebellum 8, and vermis 10. Additionally, in the SleET group relative to HCs, there were lower nodal centralities in the bilateral middle frontal gyrus (MFG) and higher nodal centralities in the left IFGtriang, right calcarine fissure, the surrounding calcarine cortex (CAL), left supramarginal gyrus (SMG), and bilateral THA. Finally, relative to the NorET group, the SleET group showed lower nodal centralities in the right MFG, right IFGtriang, left HIP, and bilateral PAL and higher nodal centralities in the right (SMA), left SMG, right PCUN, right AMYG, medial cingulate and paracingulate gyri (DCG), right cerebellum 8, and vermis 10 (Figure 2).

### Alterations of Morphological Connection Characteristics

**Figure 3:**
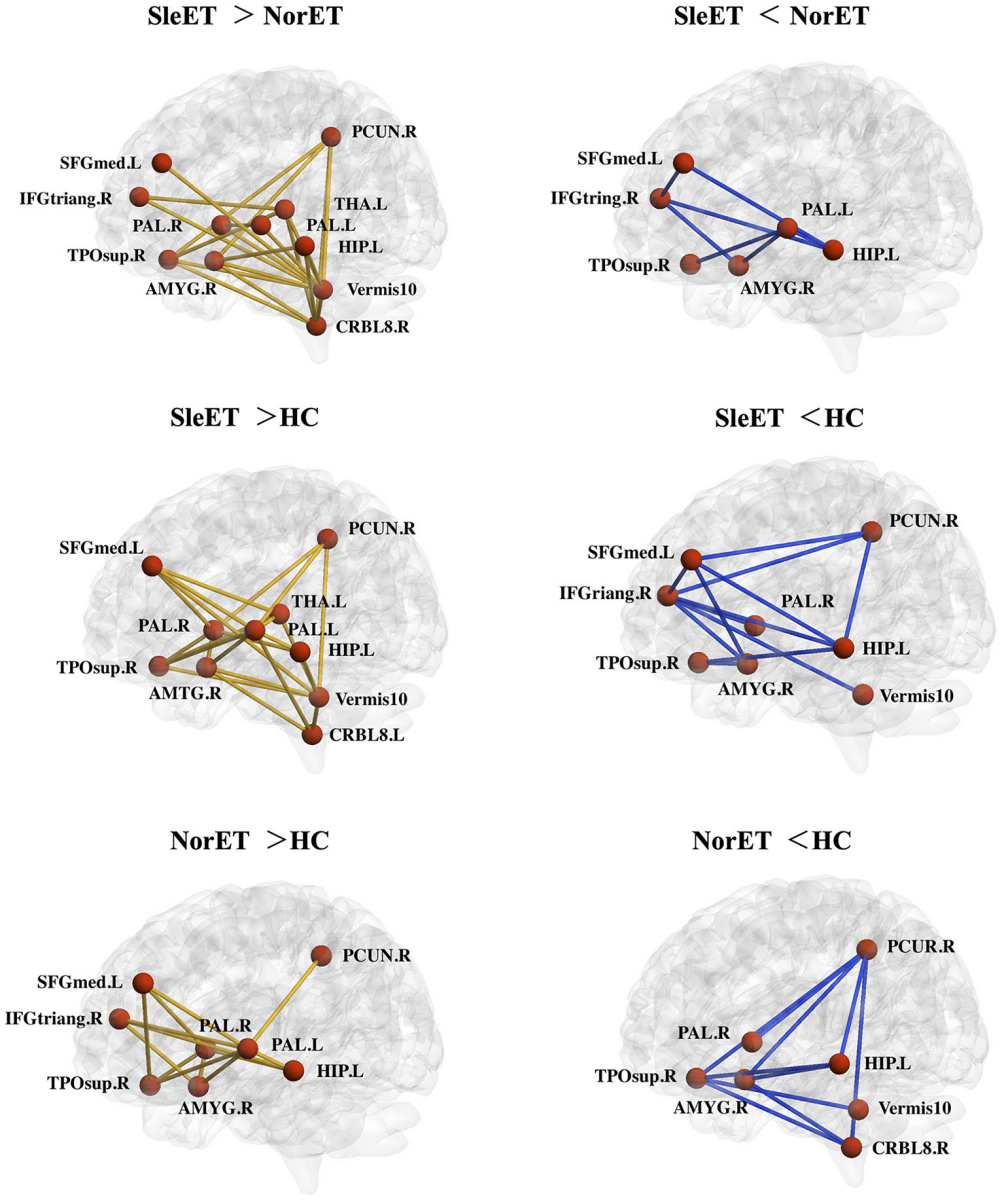
Networks showing altered morphological connections compared among the SleET, NorET, and HC groups. Red nodes denote brain regions, and lines denote connections. Yellow/blue lines represent increased/decreased morphological connections. ET, essential tremor; SleET, ET with poor sleep quality; NorET, ET with normal sleep quality; HC, healthy control; IFGtriang, inferior frontal gyrus, triangular part; SFGmed, superior frontal gyrus, medial; PCUN, precuneus; HIP, hippocampus; AMYG, amygdala; DCG, medial cingulate and paracingulate gyri; PAL, pallidum; THA, thalamus; CRBL, cerebellum; L, left; R, right.

We used the NBS tool to explore morphological network alterations in brain regions that showed significant between-group differences in nodal properties. In the SleET group relative to the NorET group, we identified two disconnected subnetworks. One subnetwork, which comprised 11 nodes and 21 increased connections, were mainly contained in the DMN (left SFGmed, left HIP, right PCUN, and right temporal pole: superior temporal gyrus [TPOsup]), the central-executive network (CEN; right IFGtriang), salience network (SN; right AMYG), cerebello-thalamo-(cortical) network (left THA, right cerebellum 8, and vermis 10), and basal ganglia network (bilateral PAL). One subnetwork comprised six nodes and seven decreased connections, which was mainly within the DMN and basal ganglia network. Furthermore, in the SleET group relative to HCs, we identified one subnetwork, comprising 10 nodes and 22 increasing connections, which was mainly within the DMN (left SFGmed, left HIP, right PCUN, and right TPOsup), SN (right AMYG), cerebello-thalamocortical network (left THA, right cerebellum 8, and vermis 10), and basal ganglia network (bilateral PAL). An additional subnetwork, comprising eight nodes and 12 decreased connections, was mainly located in the DMN and CEN (Figure 3).

### Correlations Between Network Properties and Clinical Variables

**Figure 4:**
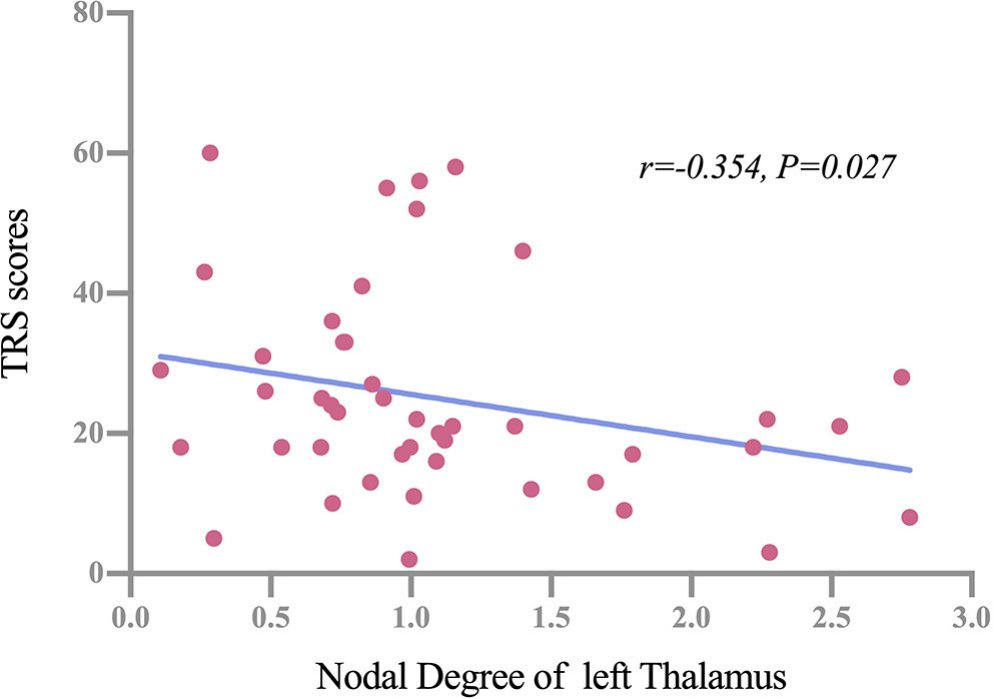
Partial correlations between the nodal degree of the left thalamus and TRS scores among patients in the SleET group. SleET, essential tremor with poor sleep quality; TRS, Fahn-Tolosa-Marin Tremor Rating Scale.

Partial correlation analysis was performed between significant GM network centralities and clinical variables (including age of onset, disease duration, TRS score, and PSQI score). Results showed that there were no significant correlations in the NorET group between significant nodal centralities and clinical variables (*p* > 0.05). In the SleET group, the nodal degree of the left THA was negatively correlated with the TRS score (*r* = −0.336, *p* =0.039; Figure 4). All results are presented in the Supplementary Tables S1, S2.

## Discussion

This Is the first structural MRI study to investigate brain morphological network alterations in patients with ET having poor QoS. We found morphological disruptions of single-subject brain networks in patients included in the SleET and NorET groups. Our findings demonstrated that (a) global network organization in patients with ET was disrupted, with patients in the SleET group exhibiting a greater disruption of topological GM network organization with a shift toward randomization than that of patients in the NorET group; (b) patients in the SleET group have a greater extent of altered nodal centralities in the IFGtriang, SFGmed, AMYG, THA, TPOsup, and cerebellum and additional altered nodal centralities in the MFG, DCG, SMG, SMA and CAL relative to HCs; (c) morphological connection alterations within the DMN and basal ganglia network were more common across the ET groups than they were to HCs and generally to a greater extent than patients in the SleET group, and alterations within the cerebello-thalamocortical network were only found among patients in the SleET group; (d) in the SleET group, the nodal degree of the left THA was negatively correlated with TRS score. The observed topological organization alterations of the GM morphological network among patients in the SleET and NorET groups extend our understanding of the underlying neural mechanisms of patients with ET having poor QoS from a morphological network perspective and may contribute to future improvements in diagnosis and treatment.

A small-world network is an optimal model between randomized and regularized networks, allowing a good balance of network segregation (reflected by *E*_loc_, *C*_p_, and λ) and integration (reflected by *E*_glob_, *L*_p_, and λ) (14). Specifically, a higher *E*_glob_ and lower *L*_p_ in the SleET group relative to the NorET group indicated a global GM network with significantly more randomization and higher integration (35). Therefore, the SleET-related changes in small-world parameters may reflect a less optimal topological organization. However, these results differ from those of several previous studies on topological functional brain networks in patients with ET and have shown that patients with ET are shifted toward weaker small-world characteristics (8, 36). Several factors may contribute to this difference. First, functional connections between regions may not be structurally connected (37). Second, connections within structural networks reflect physical connections (synapses or axonal projections) (38); thus, disruptions within such networks may trigger a reciprocal compensatory mechanism with the functional network, which may result in inconsistent results. Moreover, sleep plays an essential role in the clearance of neurotoxic waste (i.e., amyloid β peptides) (39), and a previous study of ^18^F-fluorodeoxyglucose positron emission tomography (PET) in healthy older adults showed that poor QoS is associated with a greater burden of cerebral amyloid β (40, 41). Bellesi et al. suggested that sleep is associated with increased expression of genes related to myelin formation (42). Therefore, poor QoS may negatively impact the brain structure of patients with ET and cause global disruption of their GM network. Furthermore, a surface-based morphometric study of primary insomnia patients showed increased cortical volume, rather than atrophy, in multiple brain regions, including frontal, cingulate, and fusiform cortices; moreover, the cortical thickness of these brain regions was positively correlated with PSQI score (43). In our case, the increased integration ability of the global GM network in patients with ET may contribute to increased reactivity to stimuli, which may induce a high arousal state and in turn, signify vulnerability to poor QoS.

In regard to nodal topological properties, we found altered nodal centralities in mainly the frontal regions and the DMN. Although previous neuroimaging studies of ET using volumetric structural MRI methods showed heterogeneous brain volume changes in cerebral cortical and subcortical structural regions (44), several studies identified frontal regions (including the SFG and MFG) with a decrease in volume or cortical thickness (45, 46). Notably, our results showed that the altered nodal properties within the frontal lobes exhibited a decreasing trend from HCs, patients in the NorET group, to patients in the SleET group. Correspondingly, disruption of the cognitive system, primarily within the frontal lobe, has also been shown to contribute to the pathogenesis of insomnia patients (47), and the decline in subjective QoS is correlated with frontal GMV loss (48, 49). Andre et al. suggested that slow-wave sleep disruption is associated with frontal amyloid deposition in older adults (50). In addition, apart from frontal regions, most of the brain regions with altered nodal centralities have been located in the DMN. Previous studies have reported significant changes within the DMN in patients with ET using functional (51) and structural MRI (8). Our NBS results revealed distinct network disruptions within the DMN, including the PCUN, which is a core midline structure within the DMN that plays the most critical role in this task-negative network (52). Poor QoS is related to frequent daydreaming, rumination, and mind wandering, which require the involvement of the DMN (53). Studies from insomnia patients have shown abnormal increases or imbalances in connectivity within the DMN (54). A hyperaroused state of the DMN can persist at night and possibly during sleep stages. In healthy adolescents, QoS is related to weaker intrinsic DMN functional connectivity (18), and loss of DMN integrity may be a state marker for insufficient sleep or poor QoS (55). Accordingly, it appears that altered top-down, rather than bottom-up, integration in sleep regulation contributes to the poor QoS in patients with ET. Furthermore, the pathogenesis underlying ET may interfere with networks responsible for sleep regulation, which may, in turn, cause poor QoS.

Additionally, the poor QoS of patients with ET may contribute to a decline in affective state. Our study excluded patients with substantial depressive or anxiety symptoms to minimize the possible confounding effects. As a result, the SleET group had higher HAMA and HAMD scores relative to the NorET group. The identified regions with altered nodal properties, which included the left HIP and right AMYG, both play critical roles in the emotion regulation system. Evidence has shown that insomnia patients exhibit increased AMYG responses to sleep-related stimuli relative to controls (56). Alterations in the AMYG-related network have also been found in patients with affective disorders (57); poor QoS is a common symptom of affective disorders and may negatively mediate affective behaviors (58). Moreover, it has also been reported that adequate non-rapid eye movement (NREM) sleep may proffer an anxiolytic benefit by restoring cingulate regions (59). Thus, patients in the SleET group may experience greater anxiety. However, we found no statistically significant correlation between altered nodal centralities and HAMA or HAMD scores. Therefore, the interaction between emotional scores and QoS in patients with ET may only partially contribute to the identified GM network alterations as a primary cause.

Furthermore, despite the distinct DMN connection alterations discussed above, the NBS results demonstrated that the alterations within the cerebello-thalamo-(cortical) network (also known as the “tremor network”) were greater in the SleET group than those in the NorET group. Although the pathogenesis of ET is currently unclear, functional MRI studies have consistently found altered intrinsic cerebellar and cerebello-thalamocortical connectivity (44), and magnetic resonance spectroscopy studies have also reported reduced GABAergic function in the cerebellum and thalamus (60). Remarkably, the SleET group showed significantly higher TRS and TRS-B scores than the NorET group, which indicated higher tremor severity among patients in the SleET group. Moreover, patients in the SleET group had higher age of onset than those in the NorET group, which may be associated with a faster rate of tremor progression (61). Thus, we postulate that the deterioration of QoS in patients with ET may be associated with an advanced pathology of ET. Contrary to our expectations, the partial correlation analysis failed to find any significant associations between PSQI score and topological properties in either ET group. This may be partially due to the cross-sectional design of our study. It remains unclear whether poor QoS in E patients with ET is a consequence of disease progression or a precursor to ET. Further investigations with follow-up assessments are needed to clarify how QoS develops with the progression of ET.

In the SleET group, the nodal degree of the left THA was negatively correlated with the TRS score. The THA is also part of the tremor network and plays an essential role in tremor genesis. Specifically, the tremor network primarily connects the cerebellum and primary motor cortex *via* the ventral intermedius nucleus of the THA (62). Previous studies have reported GM volume loss (6) and altered functional connectivity (63) of the THA in patients with ET. Evidence from studies using deep brain stimulation targeting the ventral intermedius nucleus of the THA and magnetic resonance-guided focused ultrasound thalamotomy for the treatment of ET suggests an interaction between the pathophysiology and the genesis of tremor within the THA (64, 65). Consequently, the altered nodal degree of the left THA in the SleET group would contribute to more severe tremor manifestation. Despite this, the THA also mediates neocortical arousal throughout the sleep-wake cycle (66). Animal experiments have demonstrated that the burst and tonic firing patterns of the centromedial THA exhibited dual control of NREM sleep-wake transitions and sleep slow waves (67). Although we did not find significant correlations between PSQI score (i.e., QoS) and nodal properties of the THA, the significant correlation between the nodal degree of the left THA and TRS score within the SleET group suggests a link between the tremor network and QoS.

There are several limitations of our study that are worth noting. First, our study used a cross-sectional design; therefore, we could not examine the progression of QoS alongside the progression of tremors or the dynamic alterations of GM networks in patients with ET. Second, although we calculated interregional similarity quantified by Kullback-Leibler divergence-based similarity to construct the GM connectivity network, which has been used widely in the exploration of morphological connectivity in various diseases, the direct biological basis of the similarity remains unclear, and additional neuroanatomical and neurophysiological evidence is needed. Third, we used the AAL algorithm for brain parcellation, which did not include subcortical regions of the locomotor network (e.g., the subthalamic nucleus). Fourth, the PSQI is a widely used tool with high sensitivity (89.6%) and specificity (86.5%) for assessing QoS (68) that is based on a subjective rather than an objective evaluation (e.g., polysomnography). Therefore, the QoS of patients with ET may have been underestimated. Fifth, the diagnosis of ET was based on the 1998 MDS criteria. Thus, the ET patients group included a mixture of pure ET and ET-plus patients. However, no significant differences were found between patients in the SleET and NorET groups when it comes to tremor distribution, tremor type, or tremor asymmetry. Nevertheless, the plus symptoms (i.e., resting tremor and impaired tendon gait) may have an impact on the results. The comparisons of the accompanying soft signs between patients in the SleET and NorET groups are provided in Supplementary Table S3. Finally, the topological analysis was based on 3D T1-weighted MRI data, and there is a lack of neuroimaging evidence for sleep disturbances in patients with ET. Future research targeting QoS of patients with ET using multimodal neuroimaging data with a longitudinal design is needed.

## Conclusion

Essential tremor patients with poor QoS exhibited disrupted topological GM network organization with a shift toward randomization. Furthermore, disrupted nodal centralities in the DMN, SN, and cerebello-thalamo-(cortical) network may have a broader impact on the QoS of patients with ET. The nodal degree of the left THA was negatively correlated with tremor severity among patients in the SleET group. Overall, this study suggests that complex interactions underlie tremor and sleep disruptions. Our findings offer valuable insight into the neural mechanisms underlying poor QoS in patients with ET.

## Data Availability Statement

The datasets generated for this study are available from the corresponding author by request.

## Ethics Statement

The studies involving human participants were reviewed and approved by the local Ethics Committee in West China Hospital, China. The patients/participants provided their written informed consent to participate in this study.

## Author Contributions

Material preparation, data collection, and analysis were performed by JP, JY, JL, DL, NL, LD, CC, QG, and RP. The first draft of the manuscript was written by JP, and all authors commented on previous versions of the manuscript. All authors read and approved the final manuscript. All authors contributed to conception and design of the study.

## Funding

This study was supported by the National Natural Science Foundation of China (81801272), Sichuan Science and Technology Program (2018HH0077), and Post-Doctor Research Project, West China Hospital, Sichuan University (2018HXBH085).

## Conflict of Interest

The authors declare that the research was conducted in the absence of any commercial or financial relationships that could be construed as a potential conflict of interest.

## Publisher's Note

All claims expressed in this article are solely those of the authors and do not necessarily represent those of their affiliated organizations, or those of the publisher, the editors and the reviewers. Any product that may be evaluated in this article, or claim that may be made by its manufacturer, is not guaranteed or endorsed by the publisher.
