# Supplementary material for: Topologically Disrupted Gray Matter Networks in Drug-Naïve Essential Tremor Patients With Poor Sleep Quality
Source: Front Neurol. 2022 Apr 26;13:834277. doi: 10.3389/fneur.2022.834277 (PMC9086904; doi:10.3389/fneur.2022.834277)
Supplement: Supplementary file 1 [file Table_1.DOCX]

**Table S1** Partial correlations of nodal centralities with clinical variables in ET with poor sleep quality.

|  |  | Age of onset | Duration | TRS | PSQI |
| --- | --- | --- | --- | --- | --- |
| **Nodal Degree** |  |  |  |  |  |
| Frontal_Mid_L | r | 0.014 | 0.011 | 0.015 | -0.025 |
|  | *p* | 0.901 | 0.921 | 0.899 | 0.815 |
| Frontal_Mid _R | r | -0.247 | 0.042 | 0.003 | -0.162 |
|  | *p* | 0.077 | 0.768 | 0.981 | 0.250 |
| Frontal_Inf_Tri_R | r | -0.132 | 0.020 | -0.281 | -0.133 |
|  | *p* | 0.424 | 0.905 | 0.083 | 0.418 |
| Frontal_Sup_Medial_L | r | -0.133 | 0.252 | -0.046 | -0.046 |
|  | *p* | 0.493 | 0.121 | 0.781 | 0.781 |
| Cingulum_Mid_L | r | 0.009 | -0.020 | 0.063 | -0.258 |
|  | *p* | 0.586 | 0.905 | 0.706 | 0.113 |
| SupraMarginal_L | r | -0.063 | -0.085 | -0.163 | -0.095 |
|  | *p* | 0.705 | 0.605 | 0.323 | 0.567 |
| Precuneus_R | r | 0.099 | -0.107 | 0.237 | -0.138 |
|  | *p* | 0.547 | 0.518 | 0.146 | 0.402 |
| Pallidum_L | r | -0.017 | -0.270 | 0.159 | -0.103 |
|  | *p* | 0.918 | 0.097 | 0.333 | 0.532 |
| Pallidum_R | r | 0.066 | -0.269 | 0.215 | 0.145 |
|  | *p* | 0.688 | 0.098 | 0.189 | 0.380 |
| Thalumus_L | r | -0.250 | -0.124 | **-0.336** | -0.176 |
|  | *p* | 0.126 | 0.453 | **0.039** | 0.283 |
| Temporal_Pole_Sup_R | r | 0.174 | -0.124 | 0.137 | 0.198 |
|  | *p* | 0.291 | 0.453 | 0.404 | 0.228 |
| Cerebellum_Crus2_R | r | 0.159 | -0.056 | -0.091 | 0.114 |
|  | *p* | 0.335 | 0.735 | 0.582 | 0.489 |
| Cerebellum_8_R | r | 0.099 | -0.236 | 0.144 | -0.243 |
|  | *p* | 0.511 | 0.148 | 0.380 | 0.136 |
| Vermis_10 | r | -0.059 | -0.032 | -0.074 | 0.088 |
|  | *p* | 0.722 | 0.846 | 0.653 | 0.593 |
| **Nodal Betweenness** |  |  |  |  |  |
| Frontal_Inf_Tri_R | r | -0.057 | 0.050 | -0.163 | -0.069 |
|  | *p* | 0.730 | 0.761 | 0.320 | 0.677 |
| Supp_Motor_Area_R | r | 0.090 | -0.134 | 0.263 | -0.036 |
|  | *p* | 0.905 | 0.091 | 0.106 | 0.826 |
| Hippocampus_L | r | 0.311 | -0.011 | 0.276 | -0.122 |
|  | *p* | 0.054 | 0.503 | 0.089 | 0.460 |
| SupraMarginal_L | r | 0.032 | -0.057 | 0.030 | -0.212 |
|  | *p* | 0.849 | 0.728 | 0.855 | 0.196 |
| Vermis_10 | r | 0.312 | -0.012 | -0.101 | -0.173 |
|  | *p* | 0.052 | 0.944 | 0.542 | 0.292 |
| **Nodal Efficiency** |  |  |  |  |  |
| Frontal_Mid_L | r | 0.156 | 0.212 | 0.205 | -0.057 |
|  | *p* | 0.343 | 0.195 | 0.210 | 0.728 |
| Frontal_Mid_R | r | 0.293 | -0.141 | 0.234 | 0.097 |
|  | *p* | 0.071 | 0.394 | 0.151 | 0.558 |
| Frontal_Inf_Oper_L | r | 0.191 | -0.119 | 0.043 | -0.172 |
|  | *p* | 0.235 | 0.157 | 0.811 | 0.296 |
| Frontal_Inf_Tri_R | r | 0.156 | -0.085 | -0.102 | -0.197 |
|  | *p* | 0.343 | 0.606 | 0.535 | 0.228 |
| Frontal_Sup_Meidal_L | r | -0.063 | 0.154 | -0.030 | 0.195 |
|  | *p* | 0.705 | 0.350 | 0.858 | 0.235 |
| Cingulum_Mid_L | r | 0.119 | -0.036 | 0.078 | 0.112 |
|  | *p* | 0.399 | 0.844 | 0.637 | 0.501 |
| Amygdala_R | r | 0.316 | -0.032 | -0.049 | 0.058 |
|  | *p* | 0.052 | 0.849 | 0.765 | 0.727 |
| Calcarine_R | r | 0.155 | -0.021 | -0.169 | -0.231 |
|  | *p* | 0.162 | 0.901 | 0.516 | 0.150 |
| Pallidum_L | r | 0.212 | -0.139 | 0.258 | 0.027 |
|  | *p* | 0.195 | 0.115 | 0.095 | 0.872 |
| Pallidum_R | r | 0.22 | -0.143 | -0.333 | 0.120 |
|  | *p* | 0.178 | 0.105 | 0.051 | 0.465 |
| Thalamus_R | r | 0.049 | 0.01 | -0.102 | -0.209 |
|  | *p* | 0.073 | 0.126 | 0.082 | 0.423 |
| Temporal_Pole_Sup_R | r | 0.205 | -0.233 | 0.162 | 0.200 |
|  | *p* | 0.210 | 0.153 | 0.323 | 0.222 |
| Cerebellum_8_R | r | 0.036 | -0.017 | -0.097 | 0.176 |
|  | *p* | 0.826 | 0.919 | 0.556 | 0.285 |
| Vermis_10 | r | 0.155 | -0.284 | 0.253 | -0.056 |
|  | *p* | 0.140 | 0.080 | 0.120 | 0.737 |

*TRS, Fahn-Tolosa-Marin tremor rating scale; MMSE, mini-mental state examination; PSQI, Pittsburg Sleep Quality Index; HAMA Hamilton anxiety rating scale; HAMD, Hamilton depression rating scale; L left, R right, Post posterior, Mid middle; Inf, inferior; Tri. triangular; Sup superior.*

*Bold numbers are statistically significant with p<0.05*
