# Supplementary material for: Topologically Disrupted Gray Matter Networks in Drug-Naïve Essential Tremor Patients With Poor Sleep Quality
Source: Front Neurol. 2022 Apr 26;13:834277. doi: 10.3389/fneur.2022.834277 (PMC9086904; doi:10.3389/fneur.2022.834277)
Supplement: Supplementary file 2 [file Table_2.DOCX]

**Table S2** Partial correlations of nodal centralities with clinical variables in ET with normal sleep quality.

|  |  | Age of onset | Duration | TRS | PSQI |
| --- | --- | --- | --- | --- | --- |
| **Nodal Degree** |  |  |  |  |  |
| Frontal_Mid_L | r | 0.003 | 0.128 | 0.015 | -0.259 |
|  | *p* | 0.981 | 0.365 | 0.899 | 0.064 |
| Frontal_Mid _R | r | -0.131 | 0.039 | 0.005 | -0.044 |
|  | *p* | 0.356 | 0.785 | 0.970 | 0.756 |
| Frontal_Inf_Tri_R | r | 0.086 | -0.092 | 0.192 | -0.044 |
|  | *p* | 0.542 | 0.515 | 0.172 | 0.759 |
| Frontal_Sup_Medial_L | r | 0.130 | -0.125 | 0.009 | 0.059 |
|  | *p* | 0.360 | 0.379 | 0.951 | 0.678 |
| Cingulum_Mid_L | r | 0.068 | -0.214 | 0.244 | 0.078 |
|  | *p* | 0.633 | 0.062 | 0.081 | 0.580 |
| SupraMarginal_L | r | -0.050 | -0.046 | -0.132 | -0.195 |
|  | *p* | 0.726 | 0.744 | 0.124 | 0.167 |
| Precuneus_R | r | 0.062 | -0.063 | 0.116 | -0.215 |
|  | *p* | 0.663 | 0.657 | 0.414 | 0.125 |
| Pallidum_L | r | 0.098 | -0.107 | 0.028 | 0.120 |
|  | *p* | 0.487 | 0.448 | 0.843 | 0.397 |
| Pallidum_R | r | 0.177 | -0.154 | -0.219 | 0.037 |
|  | *p* | 0.209 | 0.276 | 0.061 | 0.792 |
| Thalumus_L | r | 0.051 | -0.046 | -0.041 | -0.045 |
|  | *p* | 0.718 | 0.748 | 0.775 | 0.751 |
| Temporal_Pole_Sup_R | r | 0.075 | -0.087 | -0.001 | -0.141 |
|  | *p* | 0.599 | 0.541 | 0.995 | 0.318 |
| Cerebellum_Crus2_R | r | 0.028 | -0.127 | -0.239 | 0.073 |
|  | *p* | 0.843 | 0.370 | 0.088 | 0.605 |
| Cerebellum_8_R | r | -0.192 | 0.183 | -0.001 | 0.049 |
|  | *p* | 0.173 | 0.193 | 0.995 | 0.730 |
| Vermis_10 | r | 0.112 | -0.123 | -0.135 | -0.060 |
|  | *p* | 0.427 | 0.385 | 0.340 | 0.672 |
| **Nodal Betweenness** |  |  |  |  |  |
| Frontal_Inf_Tri_R | r | 0.145 | -0.120 | 0.191 | -0.129 |
|  | *p* | 0.305 | 0.396 | 0.175 | 0.363 |
| Supp_Motor_Area_R | r | 0.044 | -0.163 | 0.214 | -0.240 |
|  | *p* | 0.758 | 0.250 | 0.108 | 0.087 |
| Hippocampus_L | r | 0.122 | -0.111 | -0.035 | -0.129 |
|  | *p* | 0.388 | 0.433 | 0.805 | 0.363 |
| SupraMarginal_L | r | 0.241 | 0.019 | 0.185 | -0.020 |
|  | *p* | 0.169 | 0.893 | 0.190 | 0.887 |
| Vermis_10 | r | -0.097 | 0.085 | 0.053 | -0.132 |
|  | *p* | 0.496 | 0.548 | 0.708 | 0.353 |
| **Nodal Efficiency** |  |  |  |  |  |
| Frontal_Mid_L | r | 0.179 | 0.109 | 0.171 | -0.011 |
|  | *p* | 0.205 | 0.443 | 0.235 | 0.939 |
| Frontal_Mid_R | r | 0.112 | -0.037 | 0.193 | -0.156 |
|  | *p* | 0.429 | 0.795 | 0.171 | 0.270 |
| Frontal_Inf_Oper_L | r | 0.136 | -0.032 | 0.102 | -0.224 |
|  | *p* | 0.336 | 0.822 | 0.471 | 0.110 |
| Frontal_Inf_Tri_R | r | 0.064 | -0.075 | 0.169 | -0.281 |
|  | *p* | 0.654 | 0.598 | 0.232 | 0.053 |
| Frontal_Sup_Meidal_L | r | 0.135 | -0.129 | -0.013 | 0.057 |
|  | *p* | 0.339 | 0.360 | 0.925 | 0.687 |
| Cingulum_Mid_L | r | 0.131 | -0.106 | 0.012 | -0.045 |
|  | *p* | 0.354 | 0.191 | 0.397 | 0.751 |
| Amygdala_R | r | 0.131 | -0.153 | 0.108 | -0.232 |
|  | *p* | 0.355 | 0.277 | 0.444 | 0.061 |
| Calcarine_R | r | 0.020 | -0.034 | -0.130 | -0.102 |
|  | *p* | 0.889 | 0.809 | 0.357 | 0.147 |
| Pallidum_L | r | 0.148 | -0.137 | -0.024 | 0.222 |
|  | *p* | 0.295 | 0.333 | 0.865 | 0.114 |
| Pallidum_R | r | 0.162 | -0.115 | -0.298 | -0.020 |
|  | *p* | 0.252 | 0.418 | 0.056 | 0.886 |
| Thalamus_R | r | 0.150 | 0.084 | -0.071 | -0.027 |
|  | *p* | 0.287 | 0.553 | 0.617 | 0.852 |
| Temporal_Pole_Sup_R | r | 0.051 | -0.063 | 0.013 | -0.159 |
|  | *p* | 0.717 | 0.656 | 0.924 | 0.259 |
| Cerebellum_8_R | r | 0.073 | -0.073 | -0.099 | -0.052 |
|  | *p* | 0.606 | 0.608 | 0.483 | 0.786 |
| Vermis_10 | r | -0.090 | 0.088 | -0.066 | -0.179 |
|  | *p* | 0.525 | 0.535 | 0.640 | 0.205 |

*TRS, Fahn-Tolosa-Marin tremor rating scale; MMSE, mini-mental state examination; PSQI, Pittsburg Sleep Quality Index; HAMA Hamilton anxiety rating scale; HAMD, Hamilton depression rating scale; L left, R right, Post posterior, Mid middle; Inf, inferior; Tri. triangular; Sup superior.*

*Bold numbers are statistically significant with p<0.05*
