# Supplementary material for: Topologically Disrupted Gray Matter Networks in Drug-Naïve Essential Tremor Patients With Poor Sleep Quality
Source: Front Neurol. 2022 Apr 26;13:834277. doi: 10.3389/fneur.2022.834277 (PMC9086904; doi:10.3389/fneur.2022.834277)
Supplement: Supplementary file 3 [file Table_3.DOCX]

Table S3. Comparisons of soft signs between SleET and NorET patients

|  | SleET | NorET | p |
| --- | --- | --- | --- |
|  |  |  |  |
| Soft signs |  |  |  |
| Questionable cerebellar signs | 18(40.0%) | 15(25.4%) | 0.114 |
| Rest tremor | 17(37.8%) | 14(23.7%) | 0.135 |
| Questionable dystonia | 1(2.2%) | 2(3.4%) | 0.724 |
| Questionable cognitive impairment | 5(11.1%) | 7(11.9%) | 0.905 |
| Others | 0 | 0 | ＞0.999 |
| Multiple soft signs | 10(22.2%) | 8(13.6%) | 0.247 |

SleET, Essential tremor with poor sleep quality; NorET, Essential tremor with normal sleep quality; HC, Healthy controls.

Note: The definition of soft signs is based on the Movement Disorder Society consensus statement of tremor classification 2018^1^, questionable cerebellar signs (intention tremor or impaired tandem gait), questionable cognitive impairment (defined as MMSE total score illiterate <17, elementary education <20, middle school education or above <24).

Reference:

1. Bhatia KP, Bain P, Bajaj N, et al. Consensus Statement on the classification of tremors. from the task force on tremor of the International Parkinson and Movement Disorder Society. Mov Disord 2018;33:75-87.
